# The Advantages of Vaginal over Suprapubic Celiotomy in Certain Diseases of the Uterus and Appendages

**Published:** 1899-04

**Authors:** H. T. Williams

**Affiliations:** Rochester, N. Y., Surgeon to the Rochester City and St. Mary’s Hospitals


### BUFFALO MEDICAL JOURNAL.

VOL. XXXVIII. APRIL, 1899. No. 9.

### Original Communications.

### THE ADVANTAGES OF VAGINAL OVER SUPRAPUBIC CELIOTOMY IN CERTAIN DISEASES OF THE UTERUS AND APPENDAGES

WITH A REPORT OF SIXTY-NINE CONSECUTIVE CASES.

By H. T. WILLIAMS, M. D., Rochester, N. Y.,
Surgeon to the Rochester City and St. Mary's Hospitals.

WHILE it is true that many operations in the pelvis can best be done by abdominal section, it is equally true that there are but few operations in the pelvis that cannot be done equally as well by vaginal section. The choice of routes will always be decided according to the experience of the surgeon.

Experience shows that in certain cases of diseases of the intrapelvic organs, many operations that were formerly done only by abdominal section can now be performed much more quickly, safely and with less shock to the patient, and with much better results, both immediate and remote, by vaginal section. By the abdominal route there is more exposure in operation, more shock, more injury to the intestines and other peritoneal surfaces generally and as the peritoneum is more exposed, consequently there is greater liability to infection. Then, again, there is no visible scar; no weak point in the abdominal wall; no hernia in the vaginal incision, and less danger from adhesions to intestines and omentum.

Fecal fistulæ occurring after abdominal section are slow to heal and often become serious, while by the vaginal section in a large percentage of cases they heal kindly and quickly.

For operable cases of cancer of the uterus, the vaginal route is undoubtedly the best one for their removal, for if a cancerous uterus

1. Read before the Medical Society of the State of New York, Albany, N. Y., February 1, 1899.

has reached the stage where it cannot be removed by the vagina, it is of little use to endeavor to remove it at all.

As a rule, the surgeon who objects strongest to operations by the vaginal route is the one who has had less experience in operations by that route. No one should attempt difficult operations of vaginal section unless he has had experience in performing abdominal section, and he should always go prepared for abdominal section, if necessary; for we must bear in mind that the abdominal route can always be chosen, even after the vaginal one, without hindrance or drawback to the operation and the opening through the vagina makes a good place for drainage. We all know the dangers of removing pus tubes through an abdominal incision; how easily is the pus spilled and how dire the consequences frequently of a few drops of pus spilled into the peritoneal cavity; how much safer and easier the vaginal route when the gauze packing gives perfect drainage aided by the natural law of gravity.

Is it not frequently the case in abdominal section, in cases of pus tubes, where large abscesses on one or both sides, with the pelvic organs and intestines firmly matted together, with dense adhesions, that it is found advisable to close the abdominal incision and drain the abscess from below?

Is it not a fact that delicate women will bear an operation by vaginal section, that would have died had the abdominal route been chosen? Dermoid cysts contain a fluid which is extremely dangerous to the peritoneum. The rupture of a dermoid has frequently produced fatal peritonitis. Death has been caused by simple aspiration of them, hence they can more safely be removed per vaginam, and this route should be chosen where the diagnosis of dermoid cysts can be correctly made.

The vaginal route possesses one of the most important factors in intrapelvic surgery, viz., natural and perfect drainage. The objection so often urged against vaginal section and for exploration of the pelvic contents, that the surgeon must depend so much on the sense of touch, more so than by abdominal section, does not count for much to the surgeon, who, from experience has become skilled in work by the vaginal route; and since the method adopted by Pryor of exploring the pelvic contents by vaginal section, as much or more can be seen by the naked eye as through the ordinary abdominal incision. A brief description of this method will not be out of place.

After the ordinary antiseptic treatment of the vagina the patient is placed in the lithotomy posture, the uterus is curetted, irrigated with either sterilised water or salt solution, and its cavity wiped out with iodoform gauze. The uterus is then packed with gauze, which is left in. The cervix is grasped with vulselum forceps and the uterus pulled down, and at the same time traction is made upward in order to put the posterior vaginal wall on the stretch. The vagina is then caught with the tenaculum or mouse tooth forceps, about an inch below its attachment to the cervix. By drawing this down about half way between this point and the cervix is the place to make the incision.

The scissors is plunged through the posterior wall of the vagina and through the peritoneum, if it can be easily perforated. If the scissors is now opened and withdrawn while open, an incision large enough to introduce the index finger will be made. If the peritoneum has not been opened with the scissors, the cavity is wiped dry, and the peritoneum picked up with forceps and cut with the scissors. By passing a finger through this opening a digital examination of the pelvic contents is now easily made. If now an ocular inspection is deemed necessary, the operator introduces two fingers into the opening in the cul-de-sac, and easily tears the vaginal mucous membrane laterally.

By now introducing one of the Pean retractors and pressing it downward, he then introduces the Pean-Pryor trowel behind the uterus, and with this forcing the uterus up leaves a wide opening into the pelvic cavity. Large gauze pads or sponges can be introduced into this opening, and thus the intestines and omentum can be held out of the way. If the patient is now turned into the Trendelenburg position, the intestines and omentum are easily made to enter into the abdominal cavity; if the intestines are adherent, the adhesions can be gently broken up. In fact a very good view can be obtained of the pelvis, even though the patient is not put into the Trendelenburg position; and should the existence of pus be suspected, the patient should not be put into this position.

By this method of Pryor an opening can be made from one and one-half to nearly three inches between the retractor and the trowel, and the intestines being held out of the way, and the complete relaxation of the pelvis muscles, owing to the Trendelenburg position, as good a view of the pelvic contents can be made as through the ordinary abdominal incision, and there is less shock to the patient and no sutures are required.

On more than one occasion, by this method, have I been able to demonstrate the vermiform appendix, and in three or four cases have

I removed the appendix as easily as I could by the abdominal method and with good results, but I do not recommend this method of operating for appendicitis.

Should a vaginal hysterectomy be indicated, the exploratory vaginal incision completes part of the operation, and the operator simply has to continue his incision around the cervix when the broad ligaments are exposed.

Ovarian cysts can be easily punctured and blood clots easily removed. Small uterine fibroids can easily be removed from the surface of the uterus and pus tubes can be dealt with as the case demands. Tubal pregnancy, hydrosalpinx, broad ligament cysts, occluded tubes and pelvic adhesions can be found and treated by this method. The after-treatment consists in simply cleaning the cavity and packing with iodoform gauze. Any retrodisplacements of the uterus, after the uterus is freed of adhesions, can be replaced and held there with the gauze, and by this method I have seen many retrodisplacements permanently cured. I usually leave the first gauze in one week, sometimes longer ; then change it every three or four days, putting in less gauze each time, until the cavity is contracted and nearly healed.

The wound in the vagina heals rapidly and the scar causes no trouble. Indeed in many cases that I have examined six months afterward no scar could be found.

Dr. William M. Polk says that vaginal section is especially indicated in the following conditions : (1) A shallow and wide pelvis in a thin woman; (2) Exploration of the pelvis; (3) Visceral adhesion in true pelvis; (4) Displaced and adherent uterus; (5) Smaller ovarian cysts, especially the intraligamentous and parovarian ; (6) Smaller fibroids, especially the soft ; (7) Extrauterine pregnancy up to the seventh month and after death of fetus; (8)Pelvic hematocele; (9)Puerperal hysterectomy; (10)Acute inflammation of the appendages, with peritonitis, involving cul-de-sac; (11) Inflammatory destructive diseases of the appendages, including tuberculous disease; (12) Pelvic abscess pointing downward; (13) Conservative operations on appendages that lie in the true pelvis.

If it is not too bold an assertion I would say that it is rarely necessary to open the abdomen for the surgical treatment of all diseases of the pelvic organs due to infection. In acute inflammation of the appendages with peritonitis, an early vaginal incision frequently cuts short the localised septic pelvic infection, the woman remaining thereafter symptomatically and physiologically well.

In a personal experience of sixty-nine consecutive vaginal sections, made in a little less than two years, for a variety of diseases of the pelvic organs, I have had sixty-eight recoveries and one death; the latter being a case of pelvic abscess, in which there was a fecal fistula. The patient had been sick for a long time and was thoroughly septic; she was operated on in the country several miles from her physician; took the anesthetic badly, and considerable chloroform was required to keep her quiet. Her physician was detained with a difficult obstetric case and did not visit her again until thirty-six hours after the operation. She was left with an inexperienced nurse, who had never before cared foı a surgical case, and the patient died forty-eight hours after the operation from vomiting and exhaustion.

I regret that the limited time will not permit my giving a more complete and detailed report of these cases. I will only say that they include many cases of large and small cystic ovaries, dermoid cysts, hydrosalpinx, pyosalpinx, ruptured tubal pregnancy, both recent and those that have become thoroughly septic, fibroids, hematocele, retroversion with adhesions, hysterectomies for both fibroids and cancer, salpingitis, acute and chronic, and in a number of cases lacerated cervices and a few perineums were repaired at the same time; in three cases of pus tubes the vermiform appendix was removed at the same time through the vaginal incision.

In conclusion I would say that neither the vaginal nor abdominal route should be chosen for all cases; each route will always have its uses, and must be studied and judged according to its indications and merits.

In the report which follows I would call the attention of the reader to cases number 1, 7, 9, 11, 13, 14, 15, 17, 23, 24, 25, 27, 29, 33, 35, 39, 48, 56, 57, 65, 68, as especially demonstrating what may be done by vaginal section and he may draw his own conclusions as to the choice of routes.

Case I.—Miss C., age 35. History: bloody urine. Pleuro-pneumonia. Temperature, 105°; pulse, 140. Double pus tubes and pelvic abscess. Operation: Uterus curetted and packed with gauze. Abscess contained one quart pus; right ovary and tube removed. Left tube (ovary healthy) split open, 4 ounces of pus evacuated and cavity packed with gauze. Time of operation twelve minutes; anesthesia fifteen minutes. Chloroform as anesthetic. No ligatures. Result: Recovery. Menstruates regularly.

Case II.—Mrs. L., age 36. Lesions: Double pus tubes with adhesions. Retroflexion. Operation: Both tubes and ovaries removed. Catgut ligatures. Gauze packing. Result: Recovery; menstruation normal.

CASE III.—Mrs. Z., age 28. Lesions: Right pus tube. Left ovary cystic. Operation: Both tubes and ovaries removed. No ligatures. Gauze packing. Result: Recovery.

CASE IV.—Mrs. W., age 30. Lesions: Salpingitis. Adhesions. Retroflexion with adhesions. Right ovary cystic. Operation: Adhesions broken up, uterus replaced, right ovary removed. Catgut ligatures. Gauze packing. Result: Recovery.

CASE V.—Mrs. H., age 26. Lesions: Pelvic abscess. Operation: Right pus tube removed. (Left ovary and tube normal). No ligatures. Gauze packing. Result: Recovery; menstruates regularly.

CASE VI.—Mrs. O., age 37. Lesions: Cystic ovaries and tubes. Retroflexed uterus adherent. Operation: Both ovaries and tubes removed. Uterus replaced with gauze packing. Catgut ligatures. Result: Recovery; menstruates regularly.

CASE VII.—Mrs A., age 24. Lesions: Cyst of left broad ligament. Operation: Opened, drained and packed with gauze. Result: Recovery.

CASE.—VIII. Mrs. D., age 29. Lesions: Right ovarian cyst. Operation: Tube and ovary removed. Uterus which was retroflexed replaced. Posterior cul-de-sac packed with gauze. Result: Recovery; uterus remained in normal position.

CASE IX.—Mrs. C., age 34. History: Pelvic pain for a long time. Had been treated with tampons. Uterus curetted and dilated. No relief. Uterus movable.. Operation: Exploration. Both ovaries cystic and tubes enlarged. Right ovary and tube removed. After breaking up some slight adhesions, cyst in left ovary was punctured and 2 drachms fluid evacuated. Ovary replaced; gauze packing. Result: Recovery and relief from pain.

CASE X.—Mrs. G., age 27. History: Miscarriage one year before, followed by pelvic inflammation. Uterus quite fixed from adhesions and exudate. Operation: Adhesions broken up and uterus and ovaries freed; gauze packing. Result: Recovery and relief from symptoms.

CASE XI.—Miss F., age 23. History: Gonorrhea six months previous. Pelvic abscess; right pus tube. Operation: One pint of pus removed. Packed with gauze. No ligatures. Result: Recovery.

CASE XII.—Mrs. C., age 40. History: Lacerated cervix and cysts of both ovaries and tubes. Operation: Trachelorrhaphy and double ovariotomy and tubes removed. Packed with gauze. No ligatures. Result: Recovery.

CASE XIII., Miss B., age 30. Lesions: Right tube and ovary diseased. Several small fibroids of uterus. Retroversion with adhesions. Operations: Small fibroids and right tube and ovary removed; adhesions broken up and uterus replaced. Gauze packing; no ligatures. Result: Much improved. Gained twenty-five pounds in three months. Uterus remained in position.

Case XIV.—Mrs. S., age 38. Lesions: Large cyst of right ovary. Right pus tube had become attached to bowel and had been discharging into it for several months. Operation: Ovary removed, tube opened and packed with gauze. Fecal fistula for some months. Finally healed. No ligatures. Result: Recovery.

Case XV.—Mrs. H., age 44. History: Uterus enlarged and microscopical examination of tissue curetted from it proved it to be cancerous. Operation: Vaginal hysterectomy. Packed with gauze; catgut ligatures. Result: Recovery. No return of cancer one and one-half years afterward.

Case XVI.—Mrs. H. H., age 54. Lesions: Fibroid of uterus and double pyosalpinx. Operation: Vaginal hysterectomy. Forceps. Packed with gauze; no ligatures. Result: Rapid recovery.

Case XVII.—Mrs. D., age 26. History and lesions: Right pyosalpinx specific. Operation: Right tube and ovary removed. Time of operation twelve minutes. Gauze packing; no ligatures. Result: Recovered.

Case XVIII.—Mrs. S., age 32. History: Right pyosalpinx. Extensive adhesions. Operation: Adhesions broken up. Right tube and ovary removed. Packed with gauze; no ligatures. Result: Recovered.

Case XIX.—Mrs. P., age 34. History: Long standing pyosalpinx discharging into rectum. Peritonitis; septic; very weak and emaciated. Extensive adhesions. Operation: Both tubes and ovaries removed, adhesions broken up, opening into bowel. Fecal fistula discharged for two months, then closed. Gauze packing, no ligatures. Result: Greatly improved.

Case XX.—Mrs. L., age 38. History: Pelvic pain and menorrhagia for years. Dysmenorrhea. Operation: Uterus curetted, adhesions around tubes and ovaries broken up. Gauze packing. Result: Complete recovery.

Case XXI.—Mrs. P., age 40. Lesions: Fibroid of left ovary, size of fist. Operation: Tumor and tube removed. Packed with gauze. Result: Rapid recovery.

Case XXII.—Mrs. H., age 37. History and lesions: Double pyosalpinx and peritonitis. Extensive adhesions. Operation: Both tubes and ovaries removed. Packed with gauze; no ligatures. Result: Recovery.

Case XXIII.—Mrs. E. G., age 42. Lesions: Cancer of uterus extending into broad ligament. Operation: Hysterectomy. Considerable of broad ligament on either side removed. Both ureters dissected out of growth. Clamps. No ligatures; gauze packing. Result: Recovery. Return of growth in kidney eight months afterward.

CASE XXIV.—Mrs. E. C., age 38. History and lesions: Lacerated cervix. Retroflexion and retroversion; adhesions to rectum. Operation: Trachelorrhaphy; uterus released and replaced. Packed with gauze. Result: Cured. Uterus in normal position one year afterward.

CASE XXV.—Mrs. R. J., age 31. History and lesions: Appendicitis. Right pyosalpinx—followed miscarriage and curettement. Temperature, 105° at the time of operation. Operation: Right tube and ovary removed. By backing up intestines with gauze pads vermiform appendix was easily seen and removed by clamping and cutting off. Appendix was size of index finger, dusky red in color and about an inch of its extremity in a sloughing condition. Gauze packing. No ligatures. Result: Complete and rapid recovery.

CASE XXVI.—Mrs. T. A., age 40. Double pyosalpinx. Right tube four inches long, and one and one-half inches thick in one place; contained several ounces of pus. Operation: Both tubes and ovaries removed, uterus replaced. Gauze packing; no ligatures. Result: Complete recovery.

CASE XXVII.—Mrs. B., age 31. History: Heart lesion—mitral—for years. Large cyst of broad ligament with large hematocele into wall of cyst. Cyst in center of hematocele. Operation: Whole mass dissected out entire; size of infant's head. Appendix discovered large and filled with blood; clamped and removed. Gauze packing. Catgut ligatures. Result: Rapid recovery.

CASE XXVIII.—Mrs. S., age 41. Lesions: Large cyst of right ovary; held three pints. Operation: Removed right ovary after evacuating contents. Left ovary also removed; contained six ounces of fluid. Both tubes thickened and also removed. Adhesions broken up and uterus replaced. Packed with gauze; no ligatures. Result: Complete recovery.

CASE XXIX.—Mrs. W., age 31. History: Three months previous operated upon by another physician for repair of lacerated cervix. Pain and some fever since. Has acquired morphine habit. Several attacks of pelvic peritonitis. Uterus immovable owing to adhesions and large exudate between uterus and rectum. Operation: Adhesions broken up, uterus and tube released; large amount of lymph and broken down tissue removed. Uterus and appendages replaced. Gauze packing; no ligatures. Result: Recovery. No pain since and takes no anodynes. Is perfectly well.

CASE XXX.—Mrs. B., age 38. History and lesions: Lacerated cervix; cysts of ovaries. Retroversion; prolapse of ovaries. Operation: Trachelorrhaphy; adhesions broken up; cyst of ovaries opened and drained. Uterus and ovaries replaced. Gauze packing. Result: Recovery. Menstruation remains regular.

CASE XXXI.—Mrs. S., age 26. History: Retroversion and retroflexion—prolapse of both ovaries. Left one adherent to rectum and beneath it was a mass, size of thumb, curved in shape and three and a half inches long; felt like thickened Fallopian tube and was diagnosticated as such by a prominent gynecologist of New York a few weeks before operation. Operation: Exploration. Adhesions broken up, uterus and ovaries released and replaced; they were both healthy. The mass in pelvis was not a tube, but was beneath rectum and firmly adherent; it was not disturbed and has been diminishing in size ever since operation one and one-half years ago. Gauze packing. Result: Patient made good recovery with relief from symptoms and has gained much in strength and flesh.

CASE XXXII.—Mrs. M. S., age 23. Lesions: Cyst of both ovaries. Left contained one quart fluid. Right two ounces fluid. Operation: Both removed; clamped for five minutes. Gauze packing; no ligatures. Result: Complete and rapid recovery.

CASE XXXIII.—Mrs. M. W., age 34. History: Lacerated cervix. Cystic degeneration. Right ovary size of large hen's egg. Operation: Trachelorrhaphy twenty minutes; ovariotomy. Five and three-quarter minutes only required to make incision; remove ovary and tube and gauze packing complete. Small catgut ligatures. Result: Rapid recovery.

CASE XXXIV.—Mrs. F. W., age 30. History: Menorrhagia and metrorrhagia. Right tube and ovary adherent and thickened. Operation: Trachelorrhaphy and right tube and ovary removed. This patient went into collapse six hours after operation; salt solution and whiskey by rectum and strychnine given. No ligatures and no hemorrhage. Time of operation, thirty-five minutes. Gauze packing. Result: Made good recovery.

CASE XXXV.—Mrs. P. A., age 33. Lesions: Cystic degeneration of right ovary and tube. Found to contain many white, hard masses, which proved to be tubercular. Operation: Right ovary and tube removed. Catgut ligatures; gauze packing. Result: Rapid recovery. In perfect health ever since. Gave birth to healthy child eleven months afterward.

CASE XXXVI.—Mrs. C. M., age 30. History: Married; two children, youngest three years old. Pelvic inflammation five years ago after first labor: very sick for several weeks. Right kidney movable; cervix lacerated; laceration extending in vaginal tissue. Left ovary cystic. Operation: Curettement. Removed large amount of degenerated tissue. Trachelorrhaphy. Left ovary and tube removed. Packed with gauze. Catgut ligature. Result: Rapid recovery. Well ever since.

CASE XXXVII.—Miss F., age 23. History: Same patient mentioned as Case No. XI. Operated upon one year ago; right tube and ovary removed. Now has pus tube on left side. Operation: Tube contained three ounces of pus and left tube and ovary removed. Gauze packing, no ligatures. Result: Recovery.

Case XXXVIII.—Mrs. K. V., age 24. History: Married four years. One child two and a half years old. Missed periods for three months, then began to flow severely with discharge of shreds and clots; flow continued for two or three weeks; both ovaries enlarged, cystic and tender. Operation: Both ovaries and tubes removed. Gauze packing; catgut ligatures. Result: Complete recovery. Patient menstruates regularly since.

Case XXXIX.—Mrs. P., age 37. Lesions: Large dermoid cyst of right ovary, contained twelve ounces of mushy fluid, hair and teeth. Operation: Right tube and ovary with sac removed. Left ovary slightly cystic, but left in position. Iodoform gauze packing; catgut ligatures. Result: Complete recovery.

Case XL.—Miss C., age 23. History: Menorrhagia, dysmenorrhea; both ovaries cystic and bound down by adhesions, especially the left. Operation: Right ovary—size of hen's egg—removed. Cyst in left ovary punctured, drained and repacked. Gauze packing; no ligatures. Result: Rapid recovery.

Case XLI.—Miss C. S., age 35. History: Lacerated cervix; cysts of both ovaries. Operation: Trachelorrhaphy; double ovariotomy. Gauze packing; no ligatures. Result: Rapid recovery.

Case XLII.—Miss M. K., age 33. Lesions: Right ovary and tube cystic; uterus retroflexed. Operation: Right ovary and tube removed; uterus replaced. Gauze packing; catgut ligatures. Result: Recovered; uterus remains in position.

Case XLIII. Miss S., age 39. Lesions: Lacerated cervix; double pyosalpinx; both ovaries cystic. Operation: Trachelorrhaphy; both ovaries and tubes removed. Gauze packing; no ligatures. Result: Perfect recovery.

Case LXIV.—Mrs. H. M., age —. Lesions: Lacerated cervix and perineum; cysts of both ovaries. Operation: Trachelorrhaphy, perineorrhaphy and double ovariotomy. Very little trouble was experienced in repacking the wound without disturbing the perineum. Catgut ligatures to pedicles. Gauze packing. Result: Good recovery.

Case XLV.—Miss P., age 18. Lesions: Double pyosalpinx specific. Very small vagina. Operation: Both tubes and ovaries removed. Gauze packing; no ligatures. Result: Complete and rapid recovery.

Case XLVI.—Mrs. W. H., age 24. History: No children. Dysmenorrhea for many years; backache, pain in pelvis, unable to do but little for years. Menses profuse, both tubes and ovaries thickened, enlarged and cystic; many adhesions. Operation: Dilatation and curettement; both tubes and ovaries removed; catgut ligatures. Adhesions broken up and uterus replaced. Gauze packing. Result: Recovery; uterus remained in position.

CASE XLVII.—Mrs. Z., age 27. Lesions: Lacerated cervix, extending into vagina; retroversion and flexion, extensive adhesions to rectum and other parts; large cyst of right ovary. Operation: Dilatation and curettement; trachelorrhaphy; adhesions broken up, right ovary removed and uterus replaced; gauze packing. Result: Uneventful recovery. Uterus remained in place.

CASE XLVIII.—Mary W., age 38. History: Tubercular history; sister had fibroid tumor; father died of cancer of the liver and had Pott's disease of the spine. Had several attacks of peritonitis. Abdominal tumor felt to be adherent to the intestines; emaciated and apparently cachectic; menorrhagia and metrorrhagia. Operation: Tumor proved to be large dermoid multilocular cyst—six distinct cysts—altogether holding one quart of mushy, yellow substance, containing teeth, large piece of bone and hair; owing to adhesions sac was not removed, but opened, irrigated and packed with gauze; adhesions to uterus broken up, uterus replaced and held in position with gauze packing, Result: Uneventful recovery. Sac discharged pus for three months; finally healed. Has gained much in flesh and strength and feels quite well.

CASE XLIX.—Mrs., H. age 35. History and lesions: Menorrhagia; right ovary enlarged and cystic and bound by adhesions to the posterior part of uterus and rectum. Several small fibroids on posterior surface of uterus; right unilateral laceration of cervix with great eversion; left tube and ovary also diseased. Operation: Uterus curetted; lacerated cervix repaired, both tubes and ovaries removed and adhesions broken up; several small fibroids removed from posterior part of uterus; gauze packing. Result: Recovery; patient well ever since.

CASE L.—Miss M. D., age 42. History: Menorrhagia and constant pain in pelvic region; one year ago uterus dilated and curetted without relief. Large fibroid in posterior wall of uterus. Operation: Vaginal hysterectomy; clamps; no ligatures; gauze packing. Result: Rapid recovery.

CASE LI.—Mrs. L. S., age 35. History: Sick two years. Prominent symptoms; pain in pelvis, chills and fever. Had many physicians who gave different opinions; when she first came under observation. Temperature, 103; both ovaries cystic. Double pus tubes; left the largest; extensive adhesions to intestines. Operation: One quart pus evacuated: adhesions broken up; both tubes and ovaries removed. Catgut ligatures; gauze packing. Result: Rapid and complete recovery.

CASE LII.—Mrs. J., age 26. History: Two or three attacks of pelvic peritonitis. Uterus reflexed and adherent. Double pus tubes and ovaries. Operation: Adhesions broken up, tubes and ovaries removed. Uterus replaced. No ligatures; gauze packing. Thirty-six hours afterward temperature arose to 103 2-5°; pulse, 120. Forty-eight hours later, without removing packing, temperature dropped to 100°; pulse, 108. Next day was normal. Results: Uneventful recovery.

CASE LIII.—Miss J. J., age 27. History: Two attacks of pelvic peritonitis; double pyosalpinx and cystic ovaries. Operation: Both ovaries and tubes removed. Gauze packing. Result: Uneventful recovery.

CASE LIV.—Mrs. B., age 26. History: Two attacks of pelvic peritonitis; double pus tubes and ovaries. Operation: Both tubes and ovaries removed. Gauze packing. Uneventful recovery.

CASE LV.—Mrs. B., age 48. History: Menorrhagia and pelvic pain; double pyosalpinx with cystic ovaries. Right tube three-quarter inch diameter; left tube one inch in diameter. Operation: Both tubes and ovaries removed; adhesions broken up, uterus replaced. Gauze packing; catgut ligatures. Result: Uneventful recovery.

CASE LVI.—Mrs. W., age 38. History and lesions: Never pregnant; small vagina; uterine fibroid, size of two fists. Anemic, almost cachectic from continued flowings. Patient weighed about eighty pounds at time of operation. Operation: Vaginal hysterectomy. Clamps. Although the vagina was very small, no trouble was experienced in stretching it and removing the uterus and tumor. Gauze packing. Result: Uneventful recovery; saw her four months afterward, when her weight was 118 pounds.

CASE LVII.—Mrs. H. L., age 30. History: No children; history of tubal pregnancy four weeks previous; since then has had continued fever and chills. At the time of operation, temperature 103°, pulse rapid; is thoroughly septic. Abdominal tumor felt projecting forward from behind uterus. It gives a distinct sense of fluctuation. Operation: Upon opening posterior cul-de-sac with scissors, a gush of pus, decomposed material and clots, amounting in all to about two quarts, poured out. The odor was foul. A good-sized placenta adherent to side of uterus and intestines was removed in pieces; it was very much decomposed. Many pieces of broken-down tissue, which resembled pieces of fetus were also removed. Both tubes and ovaries were so degenerated that they were easily broken off and no ligatures applied. The cavity was mopped out with sponges and packed with iodoform gauze. Result: Uneventful recovery.

CASE LVIII.—Mrs. S. B., age 37. History: No children; sick for five years before operation; began with pain in right side, over McBurney's point. At time of operation had general peritonitis; great distention of abdomen; temperature 103 2-3°, pulse 130; bowels had not moved in three days. With one finger in vagina, pressing behind cervix, with the other hand pressing on abdomen, over McBurney's point, gave sense of fluctuation in both places. Operation: Uterus curetted; then an opening was made behind cervix; one and a half quarts of pus evacuated, some adhesions broken up, both ovaries and tubes were adherent, but otherwise healthy, were left in position. Irrigated with sterilised water; iodoform gauze packing. Result: Uninterrupted recovery.

CASE LIX.--Mrs. G., age 30. History: Two children, youngest four years old; patient had been sick for several weeks; abdominal and pelvic pain, especially in the right side. Fever; peritonitis. Operation: Curettement; posterior vaginal section; uterus found adherent to the rectum; large exudate in right side of pelvis; extensive adhesions between the uterus and tubes and exudate around the uterus. Six to eight ounces of fetid pus evacuated; adhesions were broken up; right tube and ovary were so degenerated as to be easily broken off and removed without ligatures. Vermiform appendix was found to be perforated. Large fecal concretion from the appendix escaped with the pus. Appendix had sloughed off and came away in sections; an opening was found in the intestine which was surrounded by a large exudate, about six inches above anus. This had evidently been discharging into the rectum for some time, as the edges of the opening were thickened in a funnel shape into the bowels; no ligatures, no sutures. After irrigating the opening was packed with three or four yards of iodoform gauze. Patient did not take the anesthetic well and took considerable chloroform to keep her quiet. Result: This patient was in the country ten miles from her physician, who did not see her until thirty-eight hours after the operation; she was left with an inexperienced nurse who had never seen a surgical case before, and consequently she had very little care. She died from exhaustion and persistent vomiting forty-eight hours after the operation.

CASE LX.—Mrs. W., age 24. History: Married; two children; lacerated cervix; retroversion; prolapse of both ovaries; both ovaries and tubes cystic. Operation: Curettement and trachelorrhaphy–fifteen minutes; double ovariotomy—ten minutes. Gauze packing; catgut ligatures. Result: Rapid recovery.

CASE LXI.—Mrs. G. W., age 25. History: Two children, youngest eighteen months; uterus retroflexed; both ovaries and tubes cystic. Operation: Uterus curetted; removed both ovaries and tubes; uterus replaced; gauze packing. Result: Rapid recovery; uterus remains in position.

CASE LXII.—Mrs. M. B., age 39. History: One child, 22 years old; never well since; pelvic and bearing down pain; uterine tissues so soft that the sound went through into abdominal cavity; double pus tubes with extensive adhesions. Operation: Uterus curetted; posterior vaginal section; one pint of pus removed and large quantity of necrotic tissue. Tubes and ovaries were so adherent and wrapped up in exudate that they were not removed. Cavity irrigated and packed with gauze. Result: Rapid recovery.

CASE LXIII.—Mrs. J. R., age 26. History: Two children, youngest three years old; large bilateral laceration of cervix. Uterus retroflexed and adherent; both tubes and ovaries cystic. Curettement. Trachelorrhaphy—nine sutures. Double ovariotomy; gauze packing. Result: Uneventful recovery.

Case LXIV.—Mrs. M. McC., age 36. History: Three children; abortion—induced—three months ago. Never well since. Pelvic pain and fever; severe pain in abdomen and pelvis; at times high temperature; double pyosalpinx with extensive adhesions. Operation: Curettement; both tubes and ovaries with difficulty removed, owing to extensive adhesions to intestine, omentum and uterus; ovaries and both tubes degenerated and filled with pus; in one place intestine was a very dark color; looked as though it might slough; no ligatures were used; iodoform gauze packing. Result: Twelve days after operation had discharge of fecal matter from vagina. This continued for six weeks when it entirely healed; patient has been perfectly well ever since; menstruates every month.

Case LXV.—Mrs. C. F. McB., age 33. History: Three months pregnant at the time of operation; at the time of operation, temperature 104 2-5°, pulse 130°; had had a temperature ranging from 102-105 for one week and frequent chills. Large cyst (size of hen's egg) of left ovary. Operation: Curettement, left ovary and tube; removed. Tube found to contain a few drops of pus; catgut ligatures; right tube and ovary which were normal were left. Gauze packing. Result: Next morning temperature dropped to 100°; next day was normal and remained so; patient made uninterrupted recovery.

Case LXVI.—Mrs. I. K., age 39. History: One child several years before; double pus tubes. Operation: Curettement; both tubes and ovaries removed; catgut ligatures; gauze packing; time of operation fifteen minutes. Result: Uninterrupted recovery.

Case LXVII.—Mrs. O. P. D., age 38, History: Deaf mute; three children; lacerated cervix; uterus retroflexed with adhesions; both tubes and ovaries prolapsed, but healthy. Operation: Curettement; trachelorrhaphy; adhesions broken up; uterus and ovaries replaced; gauze packing. Result: Uninterrupted recovery.

Case LXIX.—Mrs. M. H., age 28. History: Married five years; never pregnant; sickness began eight months ago; pelvic abscess had discharged into intestine; operated upon three times by another physician by incision through vagina and rubber drainage tube; since then has had pain and fever and at times discharge of fecal matter through vagina. When she came under my observation, temperature 102°, pulse rapid; vaginal section, extensive adhesions to intestines, etc. Right tube and ovary very adherent, enlarged and degenerated; ovary full of pus and pus had burrowed in different directions. Operation: Adhesions broken up; right ovary and portion of tube removed; uterus replaced, left ovary and tube freed of adhesions and left; no ligatures; gauze packing. Result: Recovery.

Case LXIX.—Mrs. H. H., age 30. History: Right pyosalpinx. Operation: Right tube and ovary removed; no ligatures; gauze packing. Result: Rapid recovery.

Just a few words as regards ligatures. In a very large number of cases I do not use them at all, simply clamping the pedicle with a stout pair of forceps and cutting, leaving the forceps on for a minute or two, then catching the pedicle with a small pair of hemostatic forceps. I remove the clamp forceps and if any bleeding is observed, clamp again, repeat this if necessary until no bleeding is observed, showing that the coats of the vessels are crushed and the vessel plugged—the gauze packing makes firm pressure and prevents hemorrhage. I have never had a case of secondary hemorrhage where no ligatures were used.

When I use ligatures I always prefer catgut and tie by Staffordshire knot, which can be applied with ease high up if necessary and easily tied by using a long piece of catgut double, holding the two ends in one hand and passing the loop over the parts to be encircled, one end of the ligature is passed through the loop by now making firm traction on both ends of the catgut; the loop can be pressed firmly into the tissues, now a simple tie twice with the ends of the catgut and the knot pressed up, the parts are firmly ligated and the ligature will not slip, the ends of the ligature can then be cut off, the pedicle grasped with forceps outside of the ligature and the tissue to be removed cut off.

52 CLINTON PLACE.

